# Intraoperative 3D imaging in plate osteosynthesis of proximal humerus fractures

**DOI:** 10.1007/s00402-023-04820-2

**Published:** 2023-03-06

**Authors:** Alexander Böhringer, Raffael Cintean, Alexander Eickhoff, Florian Gebhard, Konrad Schütze

**Affiliations:** Department of Trauma Hand and Reconstructive Surgery, Ulm University, Albert-Einstein-Allee 23, 89081 Ulm, Germany

**Keywords:** Proximal humerus fracture, PHILOS locking plate, Bone cement screw tip augmentation, Intraoperative 3D image intensifier, Digital volume tomography, Screw cutout

## Abstract

**Introduction:**

Proximal humerus fractures are common and often associated with osteoporosis in the elderly. Unfortunately, the complication and revision rate for joint-preserving surgical treatment using locking plate osteosynthesis is still high. Problems include insufficient fracture reduction and implant misplacement. Using conventional intraoperative two dimensional (2D) X-ray imaging control in only two planes, a completely error-free assessment is not possible.

**Materials and methods:**

The feasibility of intraoperative three-dimensional (3D) imaging control for locking plate osteosynthesis with screw tip cement augmentation of proximal humerus fractures was retrospectively studied in 14 cases with an isocentric mobile C-arm image intensifier set up parasagittal to the patients.

**Results:**

The intraoperative digital volume tomography (DVT) scans were feasible in all cases and showed excellent image quality. One patient showed inadequate fracture reduction in the imaging control, which then could be corrected. In another patient, a protruding head screw was detected, which could be replaced before augmentation. Cement distribution in the humeral head was consistent around the screw tips with no leakage into the joint.

**Conclusion:**

This study shows that insufficient fracture reduction and implant misplacement can be easily and reliably detected by intraoperative DVT scan with an isocentric mobile C-arm set up in the usual parasagittal position to the patient.

## Introduction

Proximal humerus fractures are common fractures in older patients with osteoporotic bone. These fractures can be a major cause of functional disability and reduction in subjective patient-perceived health [1]. If surgical intervention is necessary with the possibility of joint preservation, the locking plate is widely used and superior to conventional plate osteosynthesis [2, 3].

However, joint-preserving surgical treatment still shows a high rate of complications and revisions [4, 5]. Main risks with an overall complication rate of 24–35% include screw cutout, fracture dislocation as well as humeral head necrosis [4]. Anatomical reduction can substantially decrease the risk of postoperative failure [6]. To increase the stability of the osteosynthesis, additional strategies were developed. Augmentation of cannulated humeral head screws using polymethylmethacrylate (PMMA) bone cement or calcium composites is possible [7–9].

For intraoperative visual control of osteosynthesis, conventional two-dimensional (2D) radiographs are usually taken with a mobile C-arm image intensifier in the parasagittal position. This limits the assessability of fracture reduction and implant position. It has been described in the literature that plain radiographs of the humeral head can provide inaccurate information about the number of fragments, the exact fracture pattern, and any screw protrusion [10–14]. With modern devices, three-dimensional (3D) digital volume tomography (DVT) can be obtained intraoperatively, which would allow unrestricted assessment of osteosynthesis. The intraoperative DVT imaging in trauma surgery and orthopedics is already used in everyday practice, e.g., for the ankle joint and the spine. The possibility of using it on the shoulder has so far only been described in a few studies. The aim of this study was to demonstrate the feasibility and usefulness of intraoperative 3D DVT imaging control for locking plate osteosynthesis with screw tip cement augmentation of proximal humerus fractures with a conventional isocentric mobile C-arm image intensifier set up in the usual parasagittal position to the patient.

## Materials and methods

**Fig. 1:**
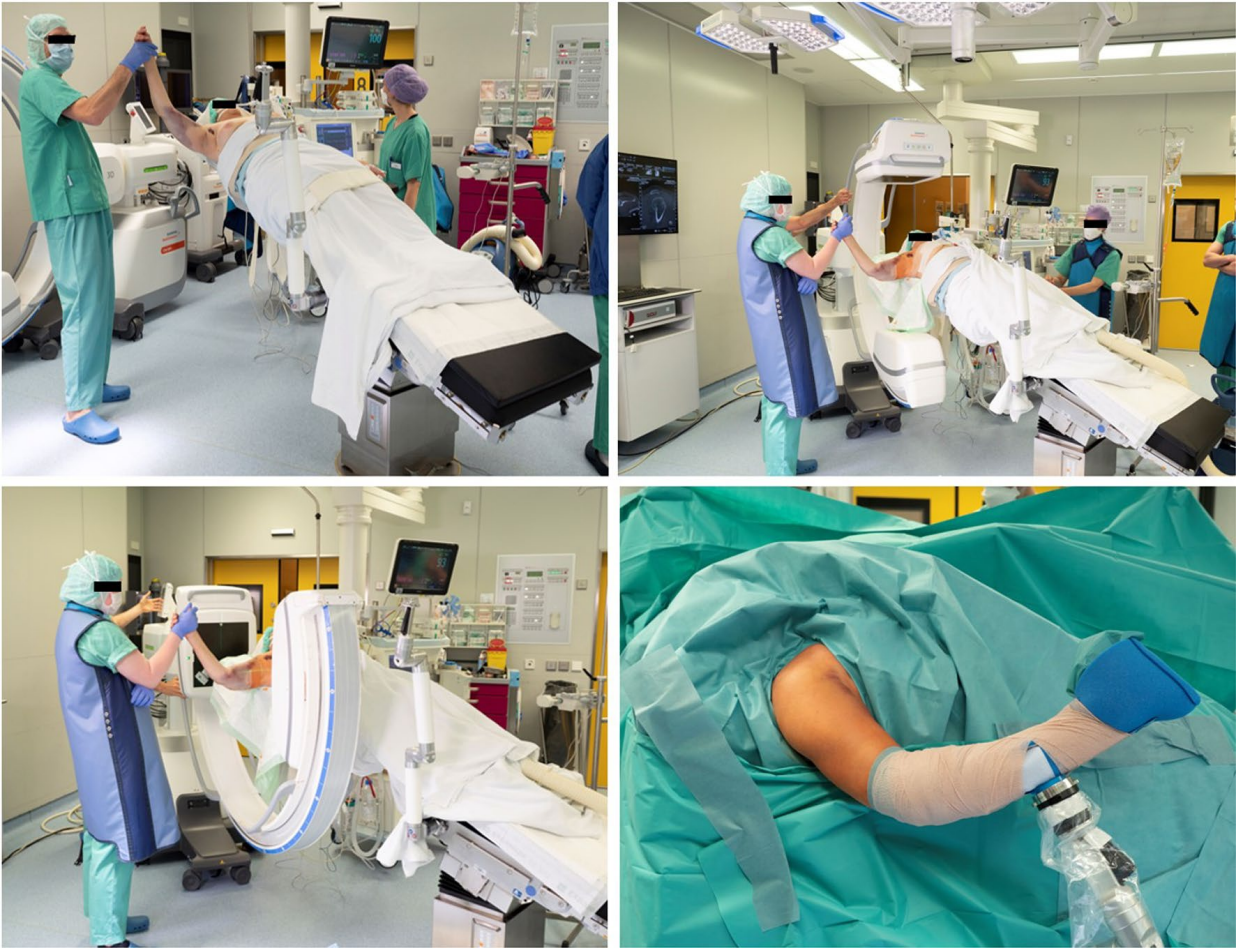
The patient in supine position on a carbon table in an assumed beach-chair position. The mobile 3D C-arm image intensifier set up in the usual parasagittal position to the patient. The patient arm mobile and fixed in the Trimano® support arm

**Fig. 2:**
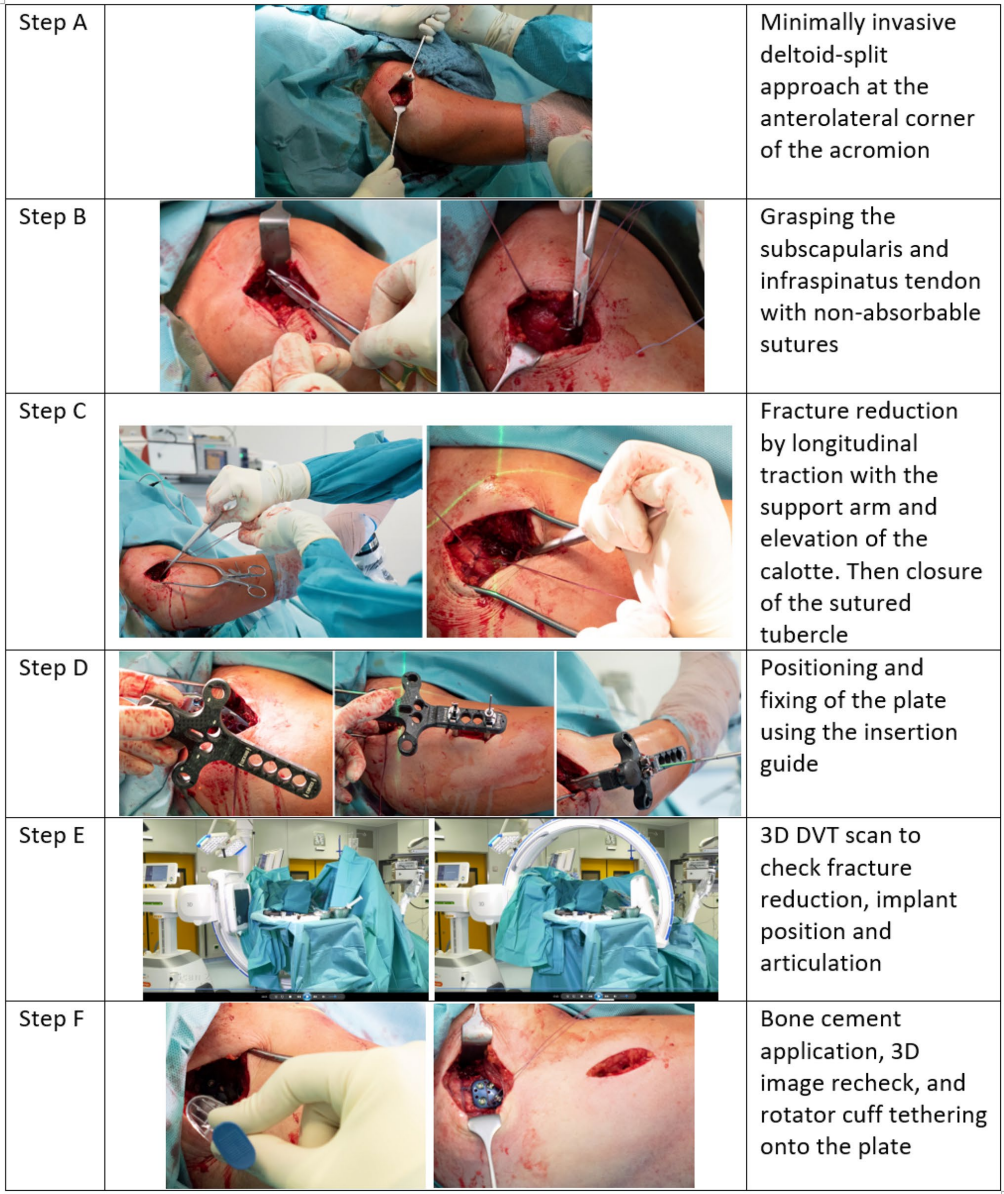
Six operation steps with (**A**) minimally invasive (MIS) deltoid-split approach, (**B**) fiber cerclage protection, (**C**) support arm reduction, (**D**) percutaneous plate aiming, (**E**) DVT imaging control and (**F**) bone cement screw tip augmentation with DVT imaging recheck

An approved ethics application for the study was obtained. Inclusion criteria were patient age > 55 years, reconstructable proximal humerus fracture, and signed informed consent. Exclusion criteria were a known infection and an existing neurovascular deficit. We chose our surgical treatment according to the four AO principles of anatomic reduction and high primary stability with a gentle approach for the earliest possible mobilization. Over 1 year, 14 patients with proximal humerus fractures and assumed osteoporosis were included. The patients were positioned supine on a carbon table in an almost beach-chair position. The isocentric mobile 3D C-arm image intensifier (Siemens Cios Spin^®^) was set up as usual parasagittal to the patient as shown in Fig. 1. The patient’s arm was freely positioned with a support arm (Arthrex Trimano^®^) and only removed and attached to the instrument table for performing the DVTs. All operations were performed by the same surgeon. The evaluation of the cases was carried out by two independent investigators. We used Charles Neer's four-segment classification system in this study [15]. It defines proximal humerus fractures by the number of displaced segments, with additional categories for articular fractures and dislocations. The four segments potentially involved are the greater tuberosity, lesser tuberosity, articular surface and humeral diaphysis. The vector of dislocation is determined by the trauma mechanism and the muscle forces of the rotator cuff attachments. In the first step (Fig. 2 Step A), a standard minimally invasive deltoid-split approach was performed. The axillary nerve was saved by leaving intact a wide soft tissue corridor at about 5–7 cm from the anterolateral acromion margin. In the second step (Fig. 2 Step B), the lesser tuberosity was fixed with sutures through the subscapularis tendon and the greater tuberosity with sutures through the infraspinatus tendon. In the third step (Fig. 2 Step C), the fracture was reduced by longitudinal traction with the support arm and elevation of the calotte fragment. The tuberosities were then tied together with the sutures like a belt. In the fourth step (Fig. 2 Step D), the locking plate (DePuy Synthes PHILOS®) was positioned through the deltoid-split approach on the proximal humerus and fixed percutaneously distal using the insertion guide. Thereafter, four bicortical shaft screws and at least four cannulated locking head screws were inserted. In the fifth step (Fig. 2 Step E), intraoperative DVT was performed with the mobile C-arm to assess fracture reduction, implant position, head–shaft position and joint articulation. Depending on the image findings, fracture reduction or implant position was improved if necessary. In the last step (Fig. 2 Step F), bone cement was applied to the cannulated screws to augment the screw tips in the humeral head. Augmentation was performed with approximately 0.5 ml of TraumacemV+^®^ bone cement (DePuy Synthes) in each case. Afterward, the second DVT scan was performed to assess the cement distribution in the humeral head and to assess possible leakage into the shoulder joint. Finally, the rotator cuff was fixed to the plate with additional non-absorbable sutures to neutralize the muscle forces.

**Fig. 3:**
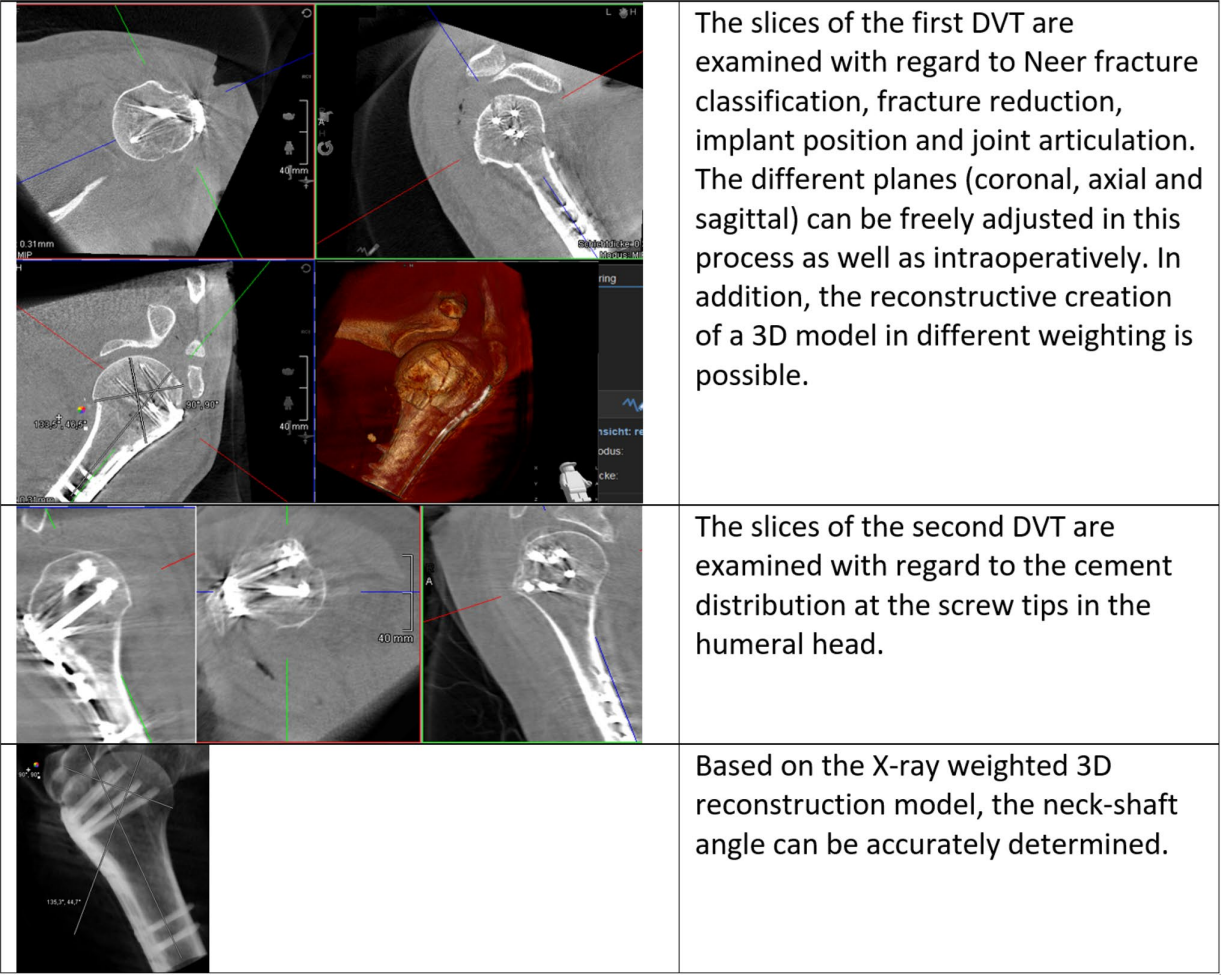
Three examples of the examination of intraoperative 3D DVT scans are presented and described. First, fracture condition, fragment reposition, implant position and joint articulation are assessed. After approval for augmentation, the cement distribution is then documented. In addition, a 3D model of the osteosynthesis can be made and measured

In the following, we illustrate the procedure with two figures and a video. Figure 1 shows the positioning of the patient and system. Figure 2 shows the step-by-step procedure during surgery. Video 1 shows the intraoperative DVT scan with the isocentric mobile C-arm image intensifier set up in the usual parasagittal position to the patient. Examples of evaluation of intraoperative 3D images for fracture classification, fragment reduction, neck–shaft angle, joint articulation, implant position and cement distribution are shown in Fig. 3.

## Results

Within 1 year, intraoperative DVT imaging was used to visually check plate osteosynthesis in 14 patients with proximal humerus fracture. Two male patients and 12 female patients suffered seven three-part and seven four-part fractures. The mean patient age was 75 years (56–89 years). All fractures were caused by low energy trauma. Osteoporosis was assumed in each case. Four patients showed additional rotator cuff (supraspinatus) tears intraoperatively. One four-part fractured shoulder was also dislocated ventrally/caudally and one four-part fracture was associated with a head split. DVT scans were feasible in all cases and showed good image quality. All examination parameters (assessment of fragment reduction, implant position, cement distribution and joint articulation) could be determined. One patient showed insufficient fracture reduction in the imaging control, which could be corrected intraoperatively. In another patient, a protruding head screw was detected prior to augmentation and was replaced with a shorter one. This screw was then not augmented. Cement distribution in the humeral head was achieved evenly around the screw tips. No cement leakage into the joint was found. The head–shaft angle was 134° on average (123–138°). The glenohumeral articulation was always centered. The results are shown in Table 1.

**Table 1:** Age and sex of the 14 patients are indicated. The always clearly possible assessment of fragment reduction, implant position, cement distribution and joint articulation is shown in the "Complete visibility" column and indicated with " + " in each case

Patient sex/age	Fracture classification	Complete visibility	Intraoperative correction	Neck–shaft angle
1 fem., 89y	3-part	+	IP	134°
2 fem., 83y	4-part	+	FR	136°
3 fem., 74y	4-part	+	−	138°
4 fem., 64y	4-part	+	−	138°
5 fem., 78y	3-part	+	−	137°
6 fem., 79y	3-part	+	−	138°
7 fem., 87y	4-part	+	−	126°
8 male, 64y	4-part	+	−	133°
9 fem., 56y	3-part	+	−	135°
10 fem., 76y	3-part	+	−	126°
11 fem., 66y	3-part	+	−	137°
12 fem., 83y	4-part	+	−	133°
13 fem., 88y	3 part	+	−	137°
14 male, 68y	4 part	+	−	123°

This also results in the respective fracture classification according to Neer (exclusively 3- and 4-part fractures) as well as the neck–shaft angle. The two one-time revision cases with fragment reduction ("FR") and implant position ("IP") are listed in the “Intraoperative correction” column

**Fig. 4:**
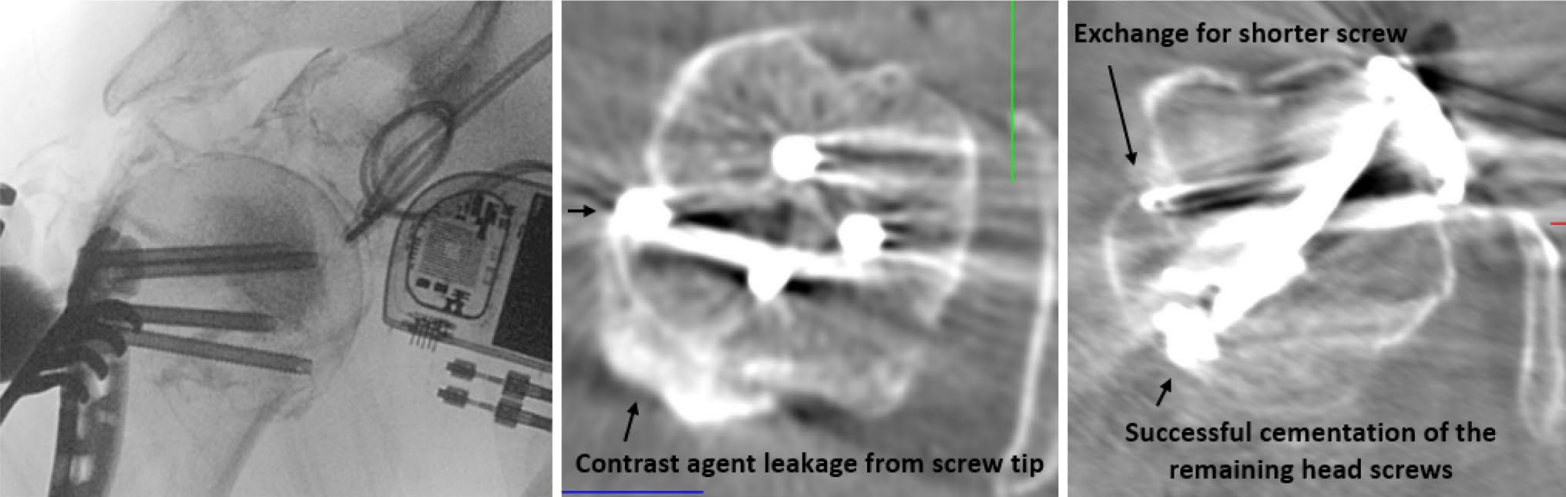
In case 1, the first DVT scan showed a screw perforation with contrast leakage into the shoulder joint. As a result, the screw in question was replaced. The remaining head screws were then successfully augmented with cement. DVT recheck showed a regular cement distribution in the humeral head without leakage into the joint

**Fig. 5:**
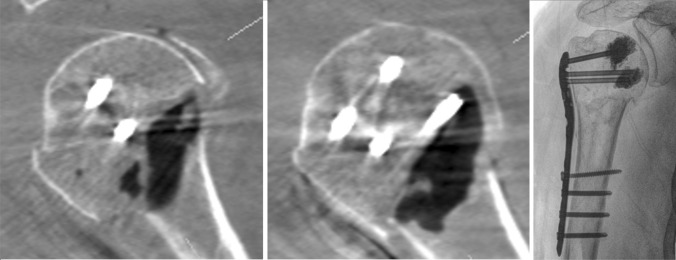
In case 2, the first DVT scan still showed a remaining dislocation of the greater and lesser tuberosity. The fracture was repositioned and the osteosynthesis material was renewed and augmented directly. The subsequent DVT scan showed satisfactory fracture reduction and implant position as well as good cement distribution

Figures 4 and 5 illustrate case 1 and 2 with one-time possible revision by an intraoperative DVT scan.

## Discussion

The aim of this study was to determine and demonstrate the feasibility and usefulness of intraoperative shoulder DVT in everyday surgical practice, using a conventional isocentric mobile C-arm set up for the first time in the usual parasagittal position to the patient.

The need to improve intraoperative imaging in the treatment of proximal humerus fractures using locking plate osteosynthesis is suggested by several studies.

In a multicenter study by Brunner et al., the incidence of implant-related complications was reported to be 9% and non-implant-related complications 35%. Primary screw perforation was the most common implant-related problem (14%), followed by secondary screw perforation (8%) and avascular necrosis (8%) [5]. A recently published systematic review of 76 studies with 4200 patients also assessed the complication rate of surgical treatment of proximal humerus fractures using the PHILOS plate [4]. With an overall complication rate of up to 29.5%, a surgical revision rate of up to 19% is reported. The most frequent complications were screw cutout, followed by fracture dislocation and humeral head necrosis as well as subacromial impingement.

When and how individual complications occur has not yet been adequately clarified or distinguished in the literature [5, 16, 17]. Thus, it remains unclear and partially inconsistent how high the proportion of primary complications (arising during the operation) and secondary complications (arising in the postoperative course) is in the whole [4, 5]. Primary complications such as insufficient fracture reduction, implant misplacement or screw cutout may not be detected by the commonly used conventional intraoperative X-ray control in two planes. Brunner et al. suggested that more accurate length measurement and shorter screw selection should prevent primary screw perforation [5]. McMillan and Johnstone even highlighted the possibility of running the drill bit left around inside the humeral head to avoid perforation into the glenohumeral joint [16]. Significant findings may also remain undetected on postoperative radiographic follow-up. However, if the postoperative conventional radiographic findings are unclear, a further CT scan must be performed. Voigt et al. [10] showed in 7 out of 30 proximal humerus fractures that the X-rays did not show the correct number of fragments. Furthermore Bahrs et al. [11] found that multiplanar diagnostics allows a significantly better assessment of the relevant structures than conventional diagnostics (*p* < 0.05) regardless of the fracture severity. In 2014, Hepp et al. [12] also showed that compared to multiplanar reconstructions, fracture morphology could not be correctly identified intraoperatively with plain radiographs in 5 out of 20 cases. These findings are in line with the studies of Theopold et al. [13, 14], who published two studies on the detection accuracy of implant perforations at the humeral head in 2017. In the first study (12 paired cadaver shoulders), subchondral Kirschner wire perforations were compared between X-ray (2D) and DVT (3D). Radiographically, at least three anteroposterior planes (true anteroposterior, 30° internal rotation and 30° external rotation) were required to detect all wire tips in each case. Using DVT, all Kirschner wires were identified correctly [13, 18]. In the second study (33 patients), fracture reduction and osteosynthesis were performed using conventional radiography. In the subsequent intraoperative 3D imaging, six malpositions were identified and corrected [14].

Thus, with intraoperative DVT control, a postoperative CT scan could be avoided.

In the humeral head, there are different areas of variable bone density, especially in the elderly. The bone density is best directly below the joint surface and in the areas of the greater and lesser tuberosity. To achieve the most stable anchoring of the implant material in the bone, the placement of the screws in these areas is aimed for. This is difficult to achieve reliably with conventional intraoperative X-ray control, and the risk of screw cutout is increased. Röderer and Scola et al. were able to show in a biomechanical study on a model that cement augmentation of the screw tips in the areas of lower bone density can significantly increase stability [19, 20]. Thus, knowledge of the areas of lower bone density as well as cement augmentation of the screws placed exactly there is important. Intraoperative 3D DVT imaging could also be beneficial in this regard.

In 2019, Theopold et al. studied 3D-assisted navigation of Kirschner wires into the glenoid center in ten patients with nonreconstructable proximal humerus fractures. They found good accuracy of the method, but the image quality in the 3D recheck with the base plate for the reverse shoulder prosthesis in place showed significant limitations due to metal artifacts [21].

In the previously published studies on intraoperative 3D DVT imaging of the shoulder, especially in the two clinical studies by Theopold et al., the mobile C-arm was set up in a transverse position to the patients in each case [12, 14, 21]. In these DVT scans, the beam path runs through the thorax from the opposite side. This probably results in a higher radiation exposure for the organs located in and adjacent to the thorax and could also result in poorer imaging of the shoulder as the target region [21, 22]. In addition, a C-arm with variable isocenter is required for transverse application, which again limits the image quality compared to conventional isocentric C-arms [22]. Furthermore, the transverse DVT scan requires a modification of the usual setup in the operating room with a more complex anesthesia position [12]. Thus, intraoperative 3D imaging of the shoulder has not yet found its way into clinical practice.

In contrast, with this study we demonstrate for the first time the feasibility of intraoperative 3D DVT imaging on the shoulder in the usual parasagittal position to the patient. The usual system setup in the OR is thus retained, saving time and effort. Furthermore, this allows imaging of the target region shoulder only and a conventional isocentric C-arm can be used. We were able to achieve good image quality with this, even with inserted implant material and PMMA cement.

## Conclusion

This study shows that insufficient fracture reduction and screw misplacement can be easily and reliably detected by intraoperative 3D DVT scan with an isocentric mobile 3D C-arm set up in the usual parasagittal position to the patient. Immediate postoperative CT examinations could thus be avoided.

## Data Availability

All authors decided that the data and material will not be deposited in a public repository.
